# The REMAR (Rhein-Main-Registry) real-world study: prospective evaluation of the 21-gene breast recurrence score® assay in addition to Ki-67 for adjuvant treatment decisions in early-stage breast cancer

**DOI:** 10.1007/s10549-024-07390-y

**Published:** 2024-06-14

**Authors:** Christian Jackisch, Louiza Anastasiadou, Sebastian Aulmann, Athanasios Argyriadis, Volker Möbus, Christine Solbach, Peter Baier, Dagmar Giesecke, Sven Ackermann, Elke Schulmeyer, Boris Gabriel, Dietrich Mosch, Stephanie Buchen, Eckart Krapfl, Ursula Hurst, Mario Vescia, Hans Tesch, Marc Thill

**Affiliations:** 1 Department of Gynecology and Obstetrics, Sana Klinikum Offenbach GmbH, Offenbach, Germany; 2 OncoNet Rhein Main e. v., Frankfurt, Germany; 3 Department of Palliative Medicine, Agaplesion Markus Hospital, Frankfurt, Germany; 4 Optipath Frankfurt MVZ Pathology, Frankfurt, Germany; 5 Department of Gynecology and Obstetrics, Städtische Kliniken Frankfurt Hoechst, Frankfurt, Germany; 6 Department of Gynecology and Obstetrics, Universitaetsklinikum Frankfurt, Frankfurt, Germany; 7 Department of Gynecology and Obstetrics, Ketteler Krankenhaus Offenbach, Offenbach, Germany; 8 Department of Gynecology and Obstetrics, Hochtaunus Kliniken, Bad Homburg, Germany; 9 Department of Gynecology and Obstetrics, Städtische Kliniken Darmstadt, Darmstadt, Germany; 10 Department of Gynecology and Obstetrics, Main Kinzig Kliniken, Gelnhausen, Germany; 11 Department of Gynecology and Obstetrics, St. Josefs Hospital, Wiesbaden, Germany; 12 Department of Gynecology and Obstetrics, Varisano Kliniken Frankfurt-Main Taunus, Bad Soden I.T., Germany; 13 Department of Obsetrics and Gynecology, Agaplesion Kliniken Wiesbaden, Wiesbaden, Germany; 14 Department of Obsterics and Gynecology, Agaplesion Klliniken Langen, Langen, Germany; 15 Department of Gynecology and Obstetrics, Kreiskrankenhaus Bergstrasse, Heppenheim, Germany; 16 Department of Obsetrics and Gynecology, GPR Klinikum Ruesselsheim, Rüsselsheim, Germany; 17 Center for Oncology and Hematology, Onkologie Bethanien, Frankfurt, Germany; 18 Department of Gynecology and Gynecological Oncology, Agaplesion Markus Hospital, Frankfurt, Germany; 19 Present Address: KEM, Evang. Kliniken Essen-Mitte gGmbH, Henricistr. 92, 45136 Essen, Germany

**Keywords:** 21-gene assay, Adjuvant treatment, Breast cancer, Ki-67, Recurrence Score,, Registry

## Abstract

**Purpose:**

Ki-67 is recommended by international/national guidelines for risk stratification in early breast cancer (EBC), particularly for defining “intermediate risk,” despite inter-laboratory/inter-observer variability and cutoff uncertainty. We investigated Ki-67 (> 10%– < 40%, determined locally) as a prognostic marker for intermediate/high risk in EBC, pN0-1 patients.

**Methods:**

This prospective, non-interventional, real-world study included females ≥ 18 years, with pN0/pN1mi/pN1, HR+ , HER2-negative EBC, and locally determined Ki-67 ranging 10%–40%. The primary outcome was changes in treatment recommendations after disclosing the Oncotype DX Breast Recurrence Score^®^(RS) assay result.

**Results:**

The analysis included 567 patients (median age, 57 [range, 29–83] years; 70%/1%/29%/ with pN0/pN1mi/pN1 disease; 81% and 19% with RS results 0–25 and 26–100, respectively). The correlations between local and central Ki-67, local Ki-67, and the RS, and central Ki-67 and the RS results were weak (*r* = 0.35, *r* = 0.3, and *r* = 0.46, respectively), and discrepancies were noted in both directions (e.g., local Ki-67 was lower or higher than central Ki-67). After disclosing the RS, treatment recommendations changed for 190 patients (34%). Changes were observed in pN0 and pN1mi/pN1 patients and in patients with centrally determined Ki-67 ≤ 10% and > 10%. Treatment changes were aligned with RS results (adding chemotherapy for patients with higher RS results, omitting it for lower RS results), and their net result was 8% reduction in adjuvant chemotherapy use (from 32% pre-RS results to 24% post-RS results).

**Conclusion:**

The Oncotype DX^®^ assay is a tool for individualizing treatments that adds to classic treatment decision factors. The RS result and Ki-67 are not interchangeable, and Ki-67, as well as nodal status, should not be used as gatekeepers for testing eligibility, to avoid under and overtreatment.

## Introduction

The sole use of classic prognostic factors to guide adjuvant treatment decisions and predict the risk of recurrence in breast cancer has been a practice for over 30 years [1–5]. The initial prognostic factors used included tumor size, nuclear and histologic grade, as well as biomarkers such as estrogen receptor (ER) and progesterone receptor (PR) status, human epidermal growth factor receptor 2 (HER2) amplification, and cathepsin D levels [1–5].

In recent years, growing evidence demonstrated that the biomarker pathways are interconnected/codependent. Therefore, assays evaluating multiple biomarkers simultaneously and weighting them appropriately, could be used to more accurately estimate the recurrence risk of an individual patient.

Among others, the 21-gene Breast Recurrence Score^®^ assay is a validated multigene assay that measures the expression of 16 cancer genes and 5 reference genes and takes into account their interconnection/codependency in the calculation of the recurrence score (RS) result from normalized gene expression levels. The RS result ranges from 0 to 100, with higher scores associated with an increased recurrence risk. Patients with high RS results were also shown to gain a substantial benefit from adjuvant chemotherapy (CT) followed by endocrine therapy (ET) [6–12]. The 21-gene assay has been used in clinical practice to adapt adjuvant treatment recommendations in patients with early-stage hormone receptor (HR) + breast cancer since 2004 [13–15].

The use of the proliferation marker Ki-67 (as determined by immunohistochemistry) as a potential guidance in adjuvant treatment decisions has been extensively investigated since it was first introduced in the 1980s, and this evolution is reflected in international guidelines. However, in spite of all attempts at standardizing the assessment of Ki-67, its use remains limited due to inter-laboratory/inter-observer variability and cutoff uncertainty [16–18]. The current US National Comprehensive Cancer Network^®^(NCCN) guidelines for breast cancer accordingly do not recommend routine assessment of Ki-67 [19]. The International Ki-67 in Breast Cancer Working Group (IKWG) published updated recommendations in 2021. These recommendations acknowledged the questionable analytical validity of Ki-67, recommended approaches to mitigate this limitation (e.g., preanalytical handling considerations, standardized visual scoring, participation in programs for quality assurance/control), and stated that in T1-2, N0-1 ER + HER2-negative patients, Ki-67 ≤ 5% or ≥ 30%, can be used to estimate prognosis [20]. The first prospective study comparing the performance of the Oncotype DX assay results and centrally determined immunohistochemistry markers including Ki-67 was the WSG Plan-B Trial in which disease-free survival (DFS) was investigated by RS result in correlation with Ki-67. The results suggested that RS results had the highest prognostic value among patients with intermediate Ki-67 levels (> 10% to < 40%) [21].

Here, we report on the Rhein-Main-Registry (REMAR) prospective, real-world German study focusing on patients with Ki-67 in the intermediate range (10–40%, as determined locally) and evaluated the reliability of this Ki-67 range as a marker to identify intermediate- or high-risk pN0-1 breast cancer patients who are likely to benefit from the prognostic/predictive information gained by the RS result. Participating clinicians were blinded to the RS results and assigned the patients to ET alone or ET plus CT (CET) based on clinicopathologic factors, including the local Ki-67 value, in their institutional multidisciplinary tumor boards, and re-evaluated the treatment decisions post-surgery when the RS results became available within a second round within the same institutional multidisciplinary tumor board.

## Methods

### Study design

The REMAR study was a prospective, non-interventional real-world cohort study conducted in the Rhein-Main region of Germany, which has a population of approximately 5.8 million people. The study included 15 certified breast cancer centers in this region.

The primary outcome of the study was the change in adjuvant treatment recommendations, as determined by the multidisciplinary tumor boards of the participating institutions, after the RS result became available. Other outcomes included the correlation between local and central Ki-67 testing and between the RS result and Ki-67 levels (as determined locally and centrally).

The study protocol was approved by the Ethics Committee at the Board of Physicians in Hesse, Germany (study number FF48/2015), and informed consent was obtained from all participating patients. The study was performed in accordance with the Declaration of Helsinki.

### Study patients and assessments

The REMAR study included female breast cancer patients ≥ 18 years, presenting with HR+ , HER2-negative, pN0/pN1mi/pN1, pT1-3, nuclear grade 1–3, and locally determined Ki-67 between 10 and 40%.

Ki-67 levels and tumor grades were evaluated both locally and centrally. The central evaluation was performed independent of the local evaluation. For central Ki-67 determination, tumor sections were sent blinded to OptiPath (S. A., Frankfurt, Germany). Ki-67 staining was performed with a ready-to-use antibody solution on Leica Bond machines (Leica Biosystems Division, Leica Microsystems Inc. Buffalo Grove, IL, United States) under standard conditions. The results were scanned with a slide scanner (3DHistech, Budapest, Hungary) and evaluated by image analysis using the ImmunoRatio software as a plugin in the ImageJ image processing package [22–24]. For each tumor, 3 representative image sections were quantified, and the mean value was calculated. 21-gene testing (Oncotype DX) was performed by Genomic Health (now part of Exact Sciences Corp., Redwood City, CA, United States) blinded for clinical and patient data. Ki-67 assessment and 21-gene testing were performed on samples from core needle biopsies.

Adjuvant treatment recommendations before and after the disclosure of the RS results were documented in a pre-specified case report form. Pre-RS, the recommended treatment was derived from histopathological (including local Ki-67) and clinical data, according to the St. Gallen consensus recommendation and the Arbeitsgemeinschaft Gynäkologische Onkologie e.V. (AGO) guidelines [25, 26]. Post-RS, treatment recommendations also incorporated the RS result. Central Ki-67 and central grading were not disclosed to the treating physicians throughout the study, and as such were not a consideration in treatment decision making.

### Statistical analysis

Descriptive statistics were used to summarize histopathological and clinical characteristics and the RS results. Pearson product-moment correlation was used to compare the RS result with local and central Ki-67.

## Results

### Patients

The study included 567 eligible patients from 15 participating centers who were diagnosed between 9/2016 and 2/2019. Baseline patient and tumor characteristics are presented in Table 1. The median age was 57 years (range, 29–83). In total 395 (70%) patients with node negative (pN0), 8 patients (1%) were staged as pN1mi. Node positivity (pN1) was found in 164 (29%) patients at diagnosis. According to local grading, 438 patients (77%) had a grade 2 tumor. According to local Ki-67 evaluation, the mean Ki-67 level was 18.8% (the range was 10–40%, as dictated by the REMAR inclusion thresholds). According to the central Ki-67 evaluation, the mean Ki-67 level was 10.3% (range; < 1%–41%).

**Table 1:** Baseline patient and tumor characteristics

Variable	*N* = 567
Age	
Median (range), years	57 (29–83)
Nodal status, *n* (%)	
pN0	395 (70%)
pN1mi	8 (1%)
pN1 (1–3 positive nodes)	164 (29%)
Tumor size	
Median (range), mm	19 (3–110)
Tumor stage (pT), *n* (%)	
1a	3 (< 1%)
1b	59 (10%)
1c	256 (45%)
2	227 (40%)
3	22 (4%)
Nuclear Grade, *n* (%)	
Central	
1	47 (8%)
2	493 (87%)
3	22 (4%)
Missing	5 (< 1%)
Local	
1	34 (6%)
2	438 (77%)
3	95 (17%)
Ki-67	
Central testing, range (mean)^a^	< 1%–41% (10.3%)
Local testing, range (mean)	10%–40% (18.8%)
Recurrence Score result, *n* (%)	
Median (range)	17 (0–61)
Recurrence Score distribution, *n* (%)	
Categories as in the initial validation studies ^2^	
0–17	303 (54%)
18–30	206 (36%)
31–100	58 (10%)
Categories as in the TAILORx and RxPONDER studies^3^	
0–25	458 (81%)
26–100	109 (19%)

^a^Ki-67 was not centrally determined for 5 patients

The median RS result was 17 (range, 0–61). The distribution of RS results using the cutoff values from the initial validation studies [6, 8, 9] demonstrated that 54%, 36%, and 10% of patients had RS results 0–17, 18–30, and 31–100, respectively. A similar analysis using an RS result cutoff value of 25 (as used in the Phase 3 validation studies TAILORx and RxPONDER [7, 10–12]) demonstrated that the majority of patients (81%) presented with a RS result between 0–25 and 19% with a RS result between 26 and 100 (Table 1).

### Ki-67 assessment—Local vs. Central

**Fig. 1:**
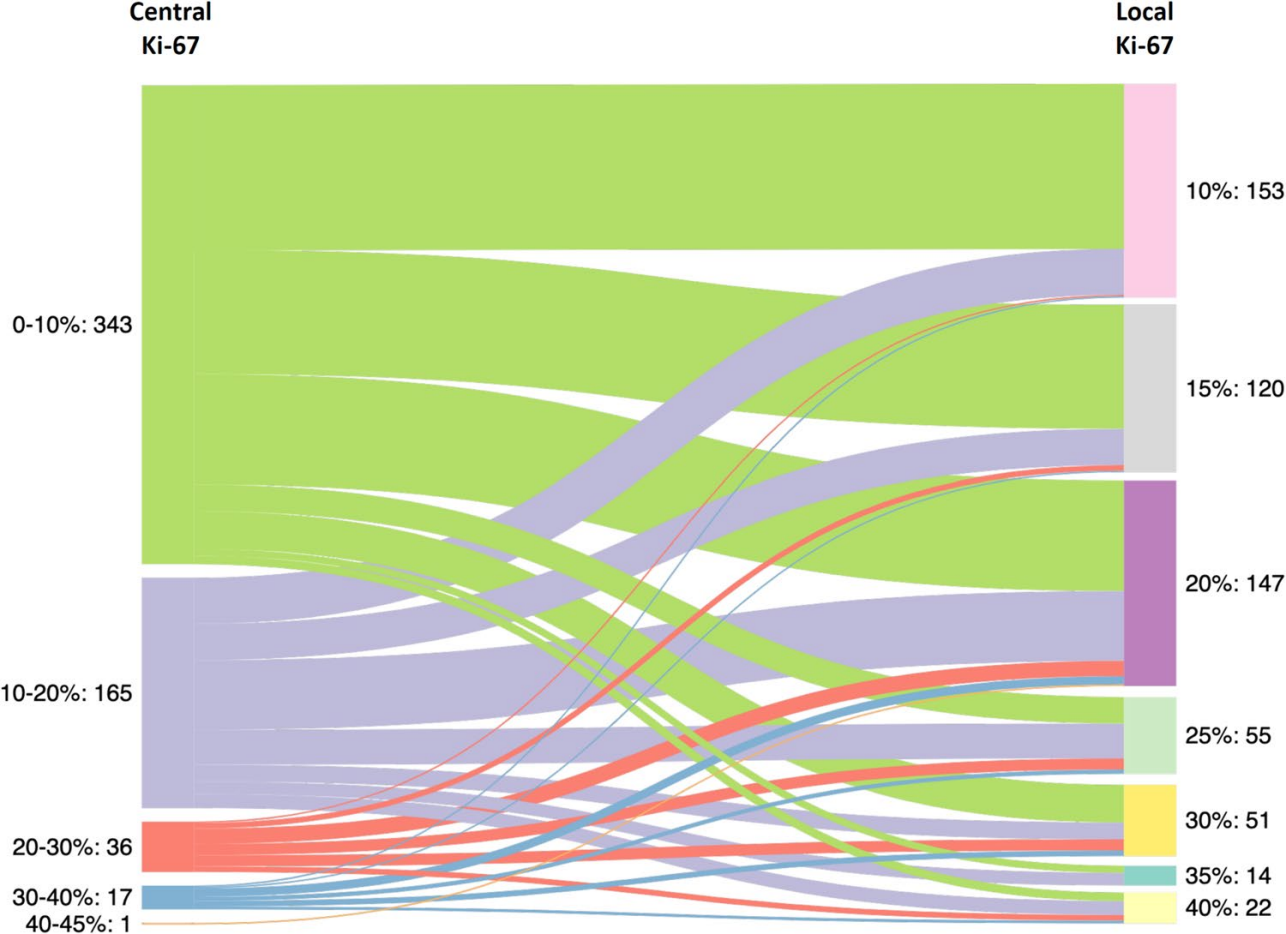
Sankey diagram displaying the correlation between Ki-67 determined centrally and locally (*n* = 562 patients with both local and central testing). The number of patients in each Ki-67 group is shown on both sides of the diagram. Local Ki-67 evaluations ranged from 10 to 40% and used routine grouping by 5% increments

The correlation between Ki-67 testing either locally or centrally (using a computerized count) is presented as Sankey flow diagram (Fig. 1). The locally assessed Ki-67 levels ranged from 10 to 40% (the inclusion threshold for REMAR) and used the routine 5% increment grouping, whereas the centrally determined Ki-67 is displayed as a continuous parameter and ranging from > 1 to 41%. The discrepancies between local and central Ki-67 evaluations were in both directions (i.e., the central Ki-67 was either lower or higher than the corresponding local evaluation). Notably, the concordance between local and central assessment was higher in low Ki-67 levels and decreased with increasing Ki-67 levels. Overall, the correlation between local and central Ki-67 levels was weak (*r* = 0.35). For 13 patients (2%), the difference in Ki-67 levels between local and central evaluations reflected a change in clinical risk and, thus, a shift in treatment recommendations if central Ki-67 would have been used to guide treatment recommendations instead of local Ki-67. These 13 patients included 12 with Ki-67_local_ > 30% and Ki-67_central_ ≤ 10% and 1 with Ki-67_local_ ≤ 10% and Ki-67_central_ > 30% (Fig. 1).

### Ki-67 and the RS results

**Fig. 2:**
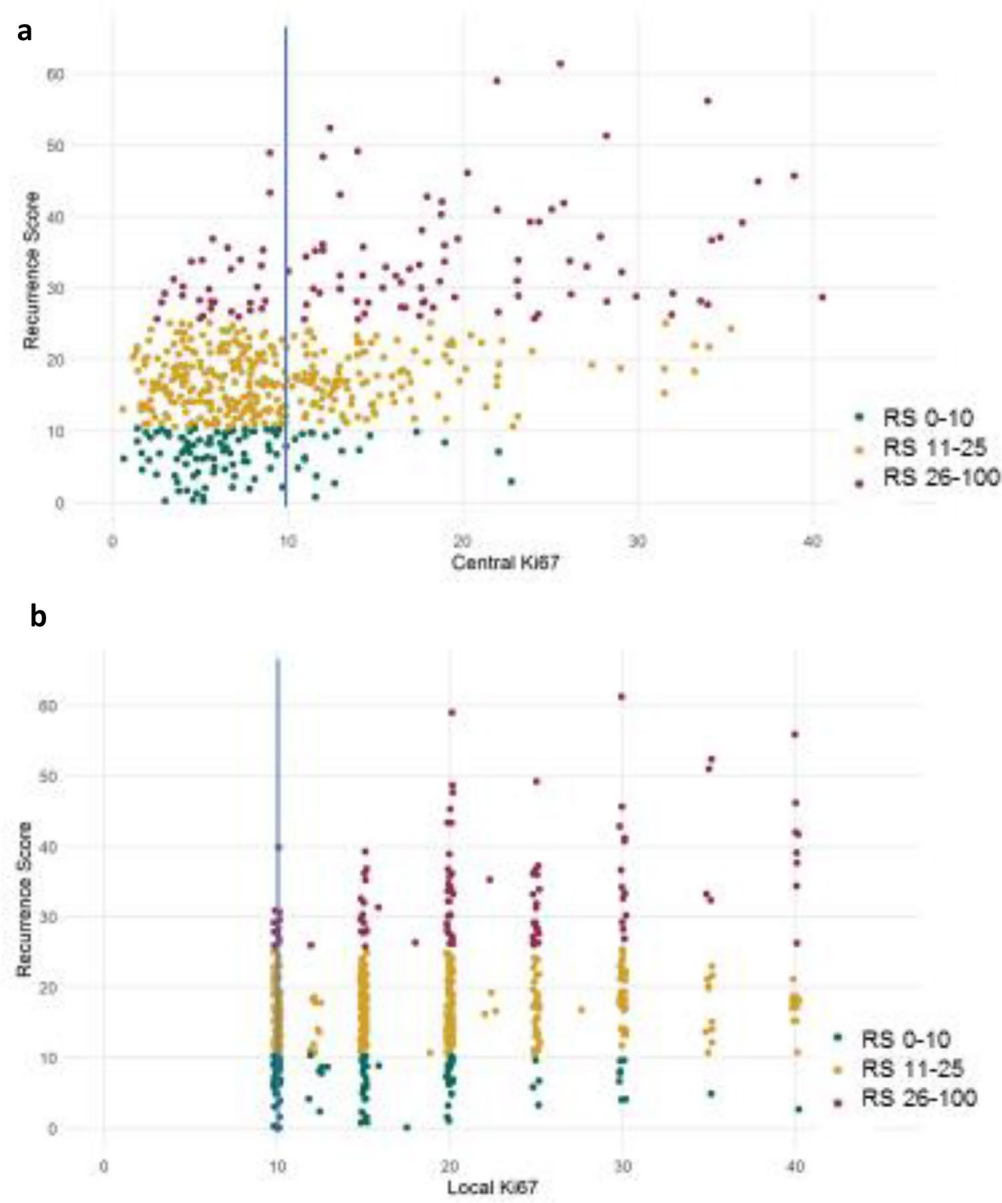
Scatter plots displaying the correlation between the RS results and Ki-67 determined centrally (**a**) or centrally (**b**). Local Ki-67 evaluations ranged from 10 to 40% per the inclusion threshold for REMAR and used routine grouping by 5% increments. The blue line represents Ki-67 levels of 10%

The correlation between the RS result and Ki-67 (local vs. central) is presented (Fig. 2). The discrepancies between RS result and central Ki-67 (Fig. 2a) as well as between RS result and local Ki-67 (Fig. 2b), as seen in the Sankey diagrams were observed in both directions. Overall, the correlation between local Ki-67 and the RS result was weak (*r* = 0.3), as was the correlation between central Ki-67 and the RS result (*r* = 0.46).

There were cases with high RS results (26–100) and low local or central Ki-67 (0–10%), as well as cases with low RS results (0–11) and high Ki-67 levels. Of the patients with central Ki-67 of 0–10% (*n* = 344), 31 (9%) had RS results 26–100. Notably, of these 31 RS result 26–100 patients, 10 were ≤ 50, and 21 were > 50 years at diagnosis. Of the patients with central Ki-67 0–20% (*n* = 504), 73 (14%) had RS result 26–100. Of these 73 RS result 26–100 patients, 25 were ≤ 50, and 48 were > 50 years at diagnosis.

### Changes in adjuvant treatment recommendations after the RS disclosure

**Fig. 3:**
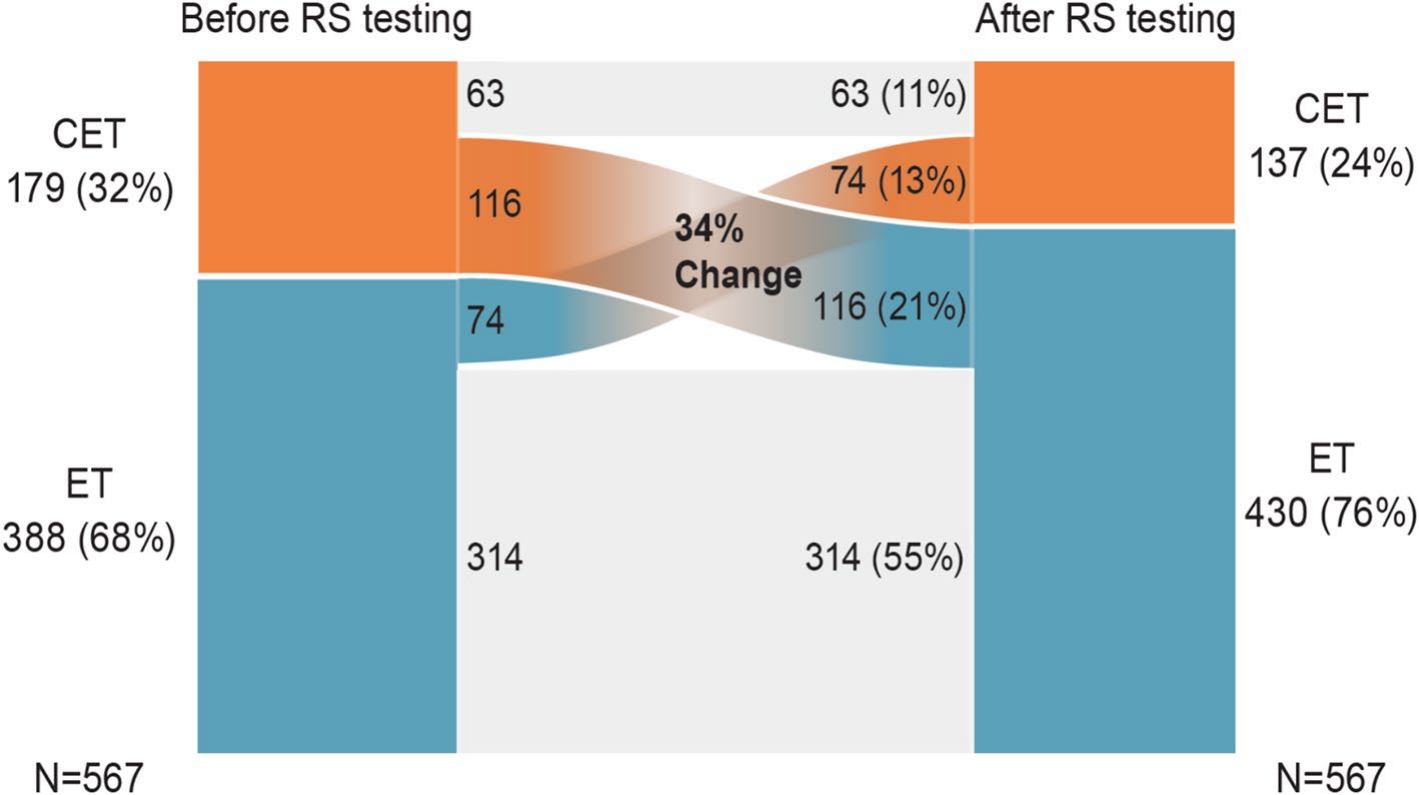
Sankey diagram displaying the absolute changes in 190 patients in treatment recommendations following the disclosure of the RS result for the entire cohort. *CET* chemoendocrine therapy, *ET* endocrine therapy, *RS* recurrence score

Absolute changes in adjuvant treatment recommendations following the disclosure of the RS result are presented for all patients (Fig. 3). Before disclosing the RS result, ET was recommended for 68% of patients and CET for 32% of patients. After disclosing the RS result, 76% of patients were recommended ET and only 24% were recommended CET (net absolute reduction in CET recommendation rate of 8%). In total, we observed a change in treatment recommendations for 190 women representing 34% of the entire population: 116 women with CET as initial therapy recommendation were transferred to ET only. In contrast, 74 individuals shifted from the initial ET group into the CET group.

Analysis of the changes in adjuvant treatment recommendations by RS result category demonstrated that treatment changes were aligned with the RS result. Among patients with RS result 0–25, treatment changes were noted in both directions but mostly involved de-escalation from CET to ET. The net effect of disclosing the RS result was an absolute reduction in CET recommendation rate of 22% (from 30 to 8%). In contrast, among patients with RS result 26–100, treatment changes were also in both directions, however, in all but one patient, these changes involved escalation from ET to CET. As expected, the net effect of disclosing the RS result in this group was an absolute increase in CET recommendation rate of 55% (from 37 to 92%) (Table 2).

**Table 2:** Treatment changes after disclosing the RS result

Pre-RS result	Post-RS result	Pre to post-RS, *n* (%)				
		CET → CET	CET → ET		ET → CET	ET → ET
RS 0–25, *n* = 458						
ET: 319 (70%)	ET: 421 (92%)	24 (5%)	115 (25%)		13 (3%)	306 (67%)
CET: 139 (30%)	CET: 37 (8%)					
RS 26–100, *n* = 109						
ET: 69 (63%)	ET: 9 (8%)	39 (36%)	1 (1%)		61 (56%)	8 (7%)
CET: 40 (37%)	CET: 100 (92%)					

*CET* chemoendocrine therapy, *ET* endocrine therapy, *RS* recurrence score

**Fig. 4:**
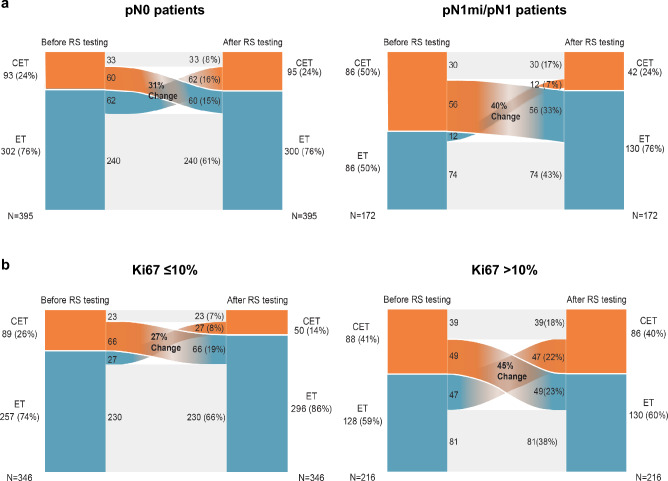
Sankey diagrams displaying the overall changes in treatment recommendations following the disclosure of the RS result by nodal status (**a**) and central Ki-67 levels (**b**). *CET* chemoendocrine therapy, *ET* endocrine therapy, *RS* recurrence score

With respect to nodal status (pN0 and pN1mi/pN1), changes of treatment recommendation in both directions (from ET to CET and from CET to ET) after disclosing the RS result were reported for a considerable proportion of the patients (31% and 40%, respectively) (Fig. 4a).

In node negative disease, changes in treatment recommendation from CET to ET occurred in 60 patients (15%) and from ET to CET in 62 individuals (16%), respectively. Thus, in absolute numbers before and after disclosing the RS results, 76% of patients were recommended ET and in 24% CET was recommended. However, in patients presenting with pN1mi/pN1 disease, representing an increased risk of recurrence, changes from CET to ET were more common than from ET to CET resulting in an absolute reduction in CET recommendation of 26% (from 50 to 24%) (Fig. 4a).

Analysis of the changes in adjuvant treatment recommendations was also performed by central Ki-67 levels using 10% as the cutoff value (Fig. 4b). This analysis examined patients with central Ki-67 ≤ 10% (*n* = 346) and patients with central Ki-67 > 10% (*n* = 216) separately. In a considerable number of patients (up to 45%), we observed recommendation changes. In patients with Ki-67 ≤ 10%, before disclosing the RS result, 74% were recommended ET and 26% were recommended CET. These recommendations were subject to change in 93 patients (27%). As de-escalation of treatment recommendation from CET to ET was more common than escalation from ET to CET, the absolute reduction in CET recommendation was 12% (from 26 to 14%). In patients with Ki-67 > 10%, before disclosing the RS result, 59% of patients were assigned to ET and CET was recommended for 41%. Changes in treatment recommendations were noted in 96 patients (45%). In 49 (23%) women, we observed a de-escalation from CET to ET and an escalation form ET to CET was observed in 47 (22%) women. The result was a net absolute reduction in CET recommendation rate of only 1% (from 41 to 40%).

## Discussion

The current study demonstrated a weak correlation between local and central Ki-67 assessment and between Ki-67 levels and the RS results. These findings are consistent with prior reports investigating Ki-67 reproducibility and inter/intra-observer variability [16–18], including findings from the prospective randomized phase 3 Plan-B study [25–33].

The current study also revealed changes in treatment recommendations that are aligned with the RS result (i.e., treatment de-escalation in RS result 0–25 patients and treatment escalation in RS result 26–100 patients), in a considerable proportion of the cohort overall (34%) and in pN0 and pN1mi/N1 patients separately (31% and 40%, respectively) with an overall net reduction in CET use. The decision-impact results are consistent with a large body of prospective data from studies conducted worldwide, which also showed such an impact on a substantial proportion of patients alongside an overall net reduction in CT use (reviewed in [34]). To the best of our knowledge, this is the first study to evaluate the impact of knowing the RS by central Ki-67 results. Most RS prospective decision-impact studies did not report the Ki-67 results of the study population. Notably, a Spanish study by Albanell and colleagues, in which the Ki-67 distribution (presumably locally determined) was reported, addressed the role of Ki-67 in treatment decision making through a univariate analysis that evaluated the association between clinicopathological variables and the likelihood of change in adjuvant treatment recommendations after knowing the RS result. The analysis demonstrated that high Ki-67 (≥ 20%) was significantly associated with a greater chance of ET to CET change, whereas a change from CET to ET was not significantly associated with Ki-67 values. Also, this univariate analysis was limited by a relatively small sample size in some of the subgroups [35].

In both the current real-world analysis, and the translational program of the Plan-B study (2274 patients with HR + HER2-negative pN0-1 early-stage breast cancer), close to a tenth of patients with low risk of recurrence according to Ki-67 level were reclassified as high risk according to the RS result (9% and 8%, respectively) [29]. A PlanB analysis which evaluated DFS by centrally determined Ki-67 groups (low: 0–10%, *n* = 790; intermediate: > 10% and < 40%, *n* = 965) and the RS result (RS: 0–11, 12–25, 26–100), showed that within the intermediate Ki-67 group, the RS was highly prognostic, whereas within the low Ki-67 group the impact of the RS was not observed. However, only 33 events occurred among all low Ki-67 patients, and only 66 patients (8.4%) had high RS [21].

Data from both the REMAR and Plan-B trials suggest that applying a Ki-67 threshold for identifying patients at intermediate risk with limiting access to the 21-gene assay might face a chance for undertreatment for a significant number of patients. Such undertreatment is of particularly relevance for young women under the age of 50, which represent about one third of our study population. As evidence suggests, these patients have a higher risk of recurrence per se. The findings also suggest that nodal status is only a weak prognosticator, as both patient subgroups (pN0 vs. pN1mi/N1) benefited from adjuvant CET [7, 10].

Following the disclosure of the RS, treatment recommendation changes were reported in both directions (escalation and de-escalation of treatment) in alignment with the RS results. Substantial change rates were observed across all the subgroups defined by nodal status, and Ki-67 level suggesting a need for a reliable clinically relevant assessment to prevent both under as well as overtreatment. Overall, the net result was a reduction in CET use; however, in node-negative disease, these treatment recommendation changes balanced out without substantial changes, at all. Notably, the decision impact of the 21-gene assay was more pronounced in the pN1mi/N1 population. More patients were recommended CET compared to the node-negative population, whereas after the assay, the proportion of patients assigned to decreased and was comparable to the use in the pN0 population (24%). Consistent with the initial validation studies in this population, as well as with the more recent phase 3 RxPONDER data demonstrating very good clinical outcomes for node-positive patients presenting with a RS result ≤ 25 treated by ET alone [7].

The REMAR results and other real-world data suggest that Ki-67 levels should not be used as eligibility criteria for initiating a 21-gene testing. For example, initially, the FDA approved abemaciclib (a CDK4/6 inhibitor) plus ET as adjuvant treatment for patients with a high-risk of recurrence in HR + HER2-negative, node-positive EBC, and Ki-67 ≥ 20% based on results from the MonarchE trial [36]. However, in 3/2021, the FDA resigned from recommending the Ki-67 level at all, based on updated data from this trial showing similar proportional invasive disease-free survival (iDFS) benefit regardless of Ki-67 status [37, 38].

Notably, the recent prospective phase 3 trial, WSG-ADAPT in HR + /HER2-negative disease suggests that Ki-67 as a dynamic biomarker after short-term neoadjuvant ET in combination with the RS result might be an additional biomarker to guide adjuvant systemic therapy decisions in pN0-1 HR + HER2-negative breast cancer. Patients received adjuvant ET if their RS result was 0–11 (control arm), or if the RS result was 12–25 and they responded to preoperative ET (experimental arm). Patients with RS result 12–25 disclosing no response to preoperative ET received CET. The ADAPT data demonstrated that the feasibility of this approach demonstrating that iDFS in the experimental arm was noninferior to the control arm. The 5-year iDFS was 92.6% (95% confidence interval [CI], 90.8–94.0%) in the experimental arm vs. 93.9% (95% CI, 91.8–95.4%) in the control arm [39, 40]. The ongoing WSG-ADAPT cycle study, a prospective, randomized phase 3 trial, investigates whether patients with HR + HER2-negative early-stage breast cancer with intermediate risk based on the RS result and response to preoperative ET (i.e., RS result ≤ 25 and Ki-67 > 10% post-induction, RS result > 25 and Ki-67 ≤ 10% post-induction in c/pN0-1 patients, or RS result ≤ 25 and Ki-67 ≤ 10% in c/pN2-3 patients) obtain additional benefit from 2 years of ribociclib (a CDK4/6 inhibitor) combined with ET compared to CT followed by ET. Interim analysis of this trial further supported the practice of combining the RS result and post-induction dynamic changes of Ki-67 for guiding adjuvant treatment and suggested that adding ovarian function suppression to tamoxifen or aromatase inhibitors (AI) improves response to ET in premenopausal patients, resulting in a response rate comparable to that of AI-treated postmenopausal patients [41].

The current study is limited by the methodology of decision-impact registries. Additionally, REMAR focused on correlations and decision-impact description and was not designed to record clinical outcome data.

## Conclusions

In conclusion, our data demonstrate in addition to the existing evidence that Ki-67 results are not validly reproducible and demonstrate a weak correlation with the RS results. Thus, the Ki-67 and the RS result are not interchangeable. Finally, the recent evidence is suggesting that Ki-67 levels should not fence the decision to indicate use of the 21-gene assay as we demonstrate like others that this test is prognostic and predictive for driving treatment decisions in luminal EBC. For the future, it is noteworthy to point out that we have to educate ourselves to leave out static considerations tools for treatment decision behind and welcome dynamic tools such as Ki-67 measurement after short-term exposure of ET to monitor endocrine sensitivity to ET. Hence, we have to welcome dynamic parameters such as dynamic Ki-67 testing as introduced in the WSG ADACT Cycle trial. The RS results provide a valuable addition to the classic risk assessment and treatment decision factors as a tool to individualize treatment decisions. Nodal status and Ki-67 alone should not be used as a gatekeeper for testing eligibility, in order to avoid under and overtreatment.

## Data Availability

The dataset used in the current study is available from the corresponding author upon reasonable request.
